# Risk factors of histologic upgrade between colposcopy-directed biopsy and loop electrosurgical excision procedure in cervical squamous intraepithelial lesions: a retrospective study

**DOI:** 10.3389/fmed.2026.1761098

**Published:** 2026-03-04

**Authors:** Yuan Ma, Bowen Xu, Tingting Zhang, Yong Zhi, Heling Ma, Yuanbo Ling, Jie Xu, Liyan Sun, Fang Li

**Affiliations:** 1 Department of Gynecology, Shanghai East Hospital, Tongji University School of Medicine, Shanghai, China; 2 Department of Gynecology, Yueyang Hospital of Integrated Traditional Chinese and Western Medicine, Shanghai University of Traditional Chinese Medicine, Shanghai, China; 3 Department of Traditional Chinese Medicine, Shanghai East Hospital, Tongji University School of Medicine, Shanghai, China; 4 Department of Gynecology, People’s Hospital of Pudong New Area, Shanghai, China

**Keywords:** cervical squamous intraepithelial lesions, colposcopy-directed biopsy, loop electrosurgical excision procedure, histolofic upgrade, HPV16/18 infection

## Abstract

**Background:**

The aim of this study is to compare the pathological outcomes between colposcopy-directed biopsy (CDB) and loop electrosurgical excision procedure (LEEP) in patients diagnosed with cervical squamous intraepithelial lesions (SIL), and to evaluate the risk factors correlated with histologic upgrade from CDB to subsequent LEEP conization.

**Methods:**

This retrospective study included a total of 1,496 patients who underwent LEEP after receiving pathological confirmation of cervical SIL through CDB. Statistical analysis was employed to assess the risk factors associated with the histologic upgrade of biopsy.

**Results:**

The study cohort was composed of 410 patients initially categorized with LSIL and 1,086 identified with HSIL, who subsequently underwent LEEP surgery. Among the LSIL-patients diagnosed via CDB, 79 showed instances of biopsy histologic upgrade. The analysis revealed that HPV16 (*p* < 0.001, *OR* 95% CI [1.58, 5.02]), HPV18 (*p* < 0.001, *OR* 95%CI [2.11, 9.29]), and type of cervical transformation zone (*p* < 0.001) were substantial risk factors leading to histologic upgrade in patients identified with LSIL by CDB. Out of the 1,086 patients who received pathological confirmation of HSIL through CDB, 19 were additionally diagnosed with cervical cancer. Among the upgraded patients, none represented type 1 cervical transformation zone. Furthermore, type 3 transformation zone was notably more susceptible to histologic upgrade compared to type 2 transformation zone (*χ*^2^ = 8.83, *p* = 0.003). Univariate analysis revealed HPV16 (*p* = 0.049, OR 95%CI [1.00, 6.58]) and HPV18 infection (*p* < 0.001, *OR* 95% CI [2.50, 18.40]) were significant contributors to the histologic upgrade of HSIL.

**Conclusion:**

Our study identified that HPV16/18 infection and a non-fully visible cervical transformation zone are significant risk factors associated with histologic upgrade between CDB and subsequent LEEP conization.

## Introduction

Cervical cancer is a prevalent gynecological malignancy, ranking fourth in both the incidence and mortality of female malignant tumors worldwide. According to the statistics in 2020, there were 604,000 newly diagnosed cases of cervical cancer globally, with 342,000 deaths (1). This population is predominantly located in developing and less developed countries. Cervical squamous intraepithelial lesion (SIL) encompasses a precancerous lesion, including low-grade cervical squamous intraepithelial lesion (LSIL) and high-grade cervical squamous intraepithelial lesion (HSIL). Approximate 80% of LSILs is characterized by the spontaneous regression, while some of them have capacity to progress to HSILs or cervical cancer (2). Thus, accurate diagnosis of female with cervical squamous intraepithelial lesions (SIL) is crucial to prevention of cervical cancer.

Persistent high-risk human papillomavirus (HR-HPV) infections are closely related to the development of precancerous lesions and cervical cancer (3). HPV screening having higher sensitivity and lower specificity compared to cytology screening, Thinprep cytology test (TCT) combined with HPV-DNA detection is commonly used for screening cervical cancer (4). Abnormal screening results require further evaluation of cervical biopsy via colposcopy (5). Colposcopy-directed biopsy (CDB) is a pivotal tool for early detection of cervical SIL and is widely regarded as the gold standard for diagnosing cervical intraepithelial neoplasia (6). Nonetheless, the diagnostic process has inherent limitations owing to the minimal tissue sample collected during biopsy. Furthermore, CDB primarily relies on the personal judgment of the colposcopy specialists, and biopsy sites may not be the most severe lesions for analysis. Some studies have reported that the accuracy of CDB in detecting SIL is suboptimal, limiting its use as a confirmatory diagnostic tool (7, 8). The cervical transformation zone (TZ) is also known as the squamocolumnar junction, as it is situated at the juncture of the squamous epithelium and the columnar epithelium of the cervix. The cervical transformation zone is classified into three types based on the visibility and location of the squamous-columnar junction under colposcopy: (1) TZ1: found at the external orifice of the cervix or outside the cervix and is entirely visible; (2) TZ2: located in the cervical canal but can be fully visualized after exposure; (3) TZ3: situated in the cervical canal and only partially visible (9). Some studies have shown that colposcopy biopsy carries the risk of histologic upgrade in SIL patients with incomplete visible cervical transformation zone (10).

It is currently considered that patients with LSIL may be closely monitored through regular follow-up visits, whereas those with HSIL require surgical intervention. Surgical approaches include cold knife conization (CKC) and loop electrosurgical excision procedure (LEEP). Conization not only serves therapeutic objectives but also allows for a definitive diagnosis of colposcopy-directed biopsy’s accuracy.

Accurate diagnosis of cervical SIL is critical for implementing appropriate management strategies, which range from active surveillance for LSIL to surgical excision for HSIL. The choice of management directly impacts patient quality of life, balancing the risks of overtreatment against those of disease progression. Therefore, evaluating and understanding the limitations of our primary diagnostic tool, CDB, by comparing it with the more comprehensive pathological assessment from excisional procedures like LEEP, is of paramount importance. Such comparisons not only enhance diagnostic accuracy but also pave the way for more targeted and individualized patient management. In present work, we compared with the differences between CDB and LEEP pathological results from 1,496 patients who were diagnosed with SIL by CDB and subsequently underwent LEEP surgery. We aimed to identify factors associated with histologic upgrade—defined as a more severe diagnosis on the LEEP specimen compared to the preceding CDB—recognizing that this discrepancy may stem from CDB sampling limitations or, less likely, interval disease progression.

## Methods

### Data collection

The patients recruited from Affiliated Hospital of Tongji University who were initially diagnosed with either LSIL or HSIL through colposcopy-directed biopsy and who subsequently underwent LEEP surgery within a 3-month span. The study period spanned from May 2014 to February 2018. Patients were further excluded from the study if they exhibited any of the following characteristics: (1) they had received cytological tests during pregnancy; (2) the time interval between their colposcopy-directed biopsy and LEEP surgery exceeded 3 months; or (3) they had previous medical histories of LEEP surgery before the study period. A limitation of the retrospective design is that the exact dates of the CDB and LEEP procedures were not available for all patients, preventing a precise calculation of the intervention interval and a sensitivity analysis based on shorter timeframes (e.g., ≤6 weeks).

### Cervical cytology test

ThinPrep Cytology Test (TCT) was utilized to collect exfoliated epithelial cells from both the cervical surface and cervical canal. The diagnosis was conducted by two pathologists specialized in cervix cytology, following the 2001 cervical cytology classification system known as The Bethesda System (TBS) (11) Cytologic diagnoses were classified as follows: negative for intraepithelial lesion or malignancy (NILM), Squamous cell abnormalities include atypical squamous cells of undetermined significance (ASCUS), atypical squamous cells-cannot exclude HSIL (ASC-H), low-grade squamous intraepithelial lesion (LSIL), high-grade squamous intraepithelial lesion (HSIL), squamous cell carcinoma (SCC) and atypical glandular cells (AGC).

### HPV genotyping test

HPV genotyping was performed using polymerase chain reaction (PCR), DNA hybridization, and an HPV genotyping DNA chip from Decipher Bioscience. The chip was designed to identify 15 high-risk HPV (16, 18, 31, 33, 35, 39, 45, 51, 52, 53, 56, 58, 59, 66, and 68).

### Colposcopy-directed biopsy

Women with abnormal TCT or HPV screening results underwent colposcopy examinations by colposcopy specialists with over 10 years of experience, following the 2012 American Society for Colposcopy & Cervical Pathology (ASCCP) guidelines. Indications for colposcopy examination included positive HPV16/18 results, persistent positive results for other types of high-risk HPVs over 1 year, and abnormal cytology findings. Biopsies were performed on suspicious lesions, and in the absence of such lesions or abnormal areas, biopsies were routinely conducted at 3, 6, 9, and 12 o’clock positions. Endocervical curettage (ECC) was performed in all cases.

### LEEP surgery

LEEP conization was performed in patients diagnosed with LSIL persistent for over 1 year and in those diagnosed with HSIL by CDB. The scale of LEEP was determined based on the lesion’s extent, the type of the transformation zone, and the patient’s reproductive wishes.

### Histopathological examination

All specimens were processed following a standardized protocol. One pathologist initially interpreted all pathological specimens, which were then verified by the second pathologist. Triage was performed on all CIN2 with p16 IHC staining, LSIL included CIN1 and P16 negative CIN2, and HSIL included P16 positive CIN2 and CIN3. Histologic upgrade was defined as a more severe diagnosis on the LEEP specimen compared to the preceding CDB. We acknowledge that this discrepancy may arise primarily from a sampling error during CDB given the limited tissue obtained, or, less likely, from true disease progression in the interval between procedures. While LEEP provides a more comprehensive specimen for evaluation, it is not a perfect reference standard, as the most severe lesion could theoretically be entirely removed by the prior biopsy. Our analysis aims to identify clinical factors associated with this clinically observed upgrade.

### Statistical analysis

SPSS 21.0 software (IBMCorp., Armonk, N. Y., USA) was used for *t*-test, chi-square test and logistic regression analysis. Variables with a *p*-value < 0.10 in the univariate analysis, or those deemed clinically relevant based on existing literature, were included in the initial multivariate logistic regression model. A backward stepwise elimination procedure was then used to retain variables with *p* < 0.05 in the final multivariate model.

## Results

### Baseline characteristics divided by CDB pathology results

**Figure 1:**
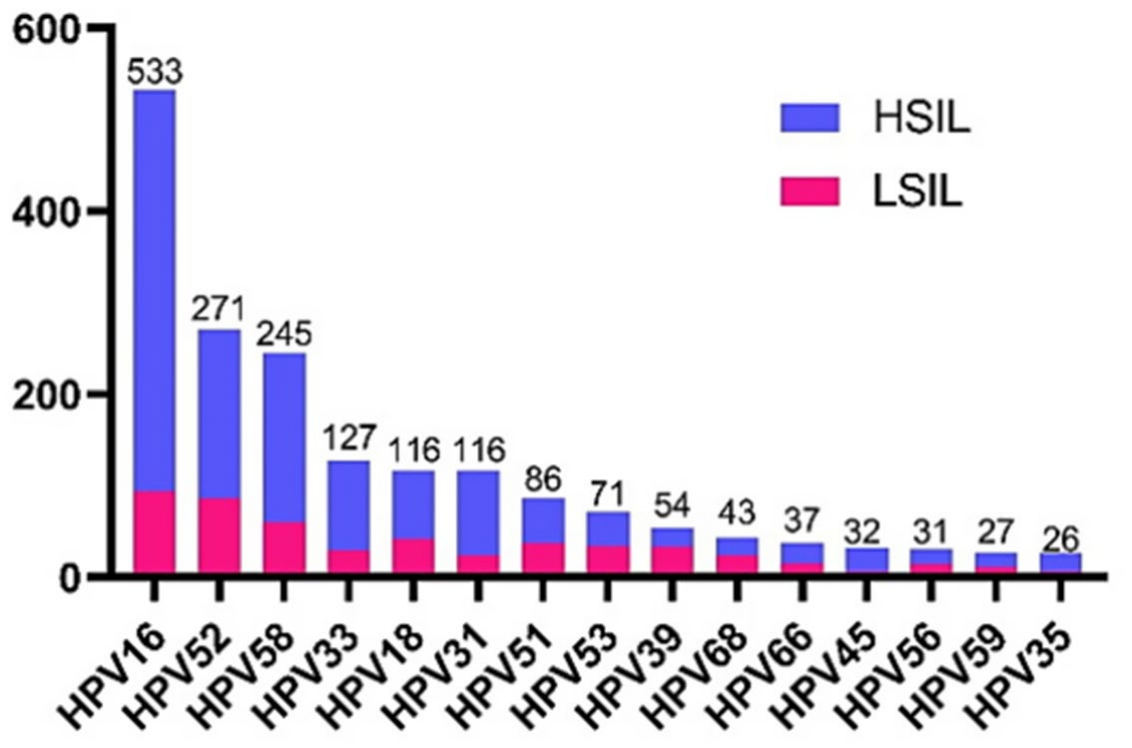
Distribution of 15 high-risk HPV infections in 1,496 cases of cervical squamous intraepithelial lesion. Pink represents the number of infections for LSIL and blue represents the number of infections for HSIL.

A total of 1,496 patients, who received a diagnosis of cervical SIL through CDB pathology and proceeded with LEEP within a span of 3 months, were recruited for this study. The study cohort included 410 patients initially diagnosed with LSIL and 1,086 patients with HSIL. The patient characteristics are presented in Table 1. The average age of all patients was 39.38 ± 9.70 years. In terms of the cervical transformation zone (TZ), 31.75, 37.43, and 30.82% of patients were identified with type 1, 2, and 3, respectively. Among the 1,496 patients, 1,321 cases (88.30%) had HR-HPV infection, with 973 (65.04%) of them infected by a single HPV subtype and 348 (23.26%) by multiple HPV subtypes. The three most prevalent HR-HPV genotypes were HPV16 (35.63%), HPV52 (17.25%), and HPV58 (16.38%), in descending order. They were followed by HPV33 (8.49%), HPV18 (7.75%), HPV31 (7.75%) (Figure 1). Out of the 1,203 women who had cytologic results available, 970 (64.84%) patients were diagnosed with NILM, ASCUS, ASC-H or LSIL. The rest of 233 (15.57%) patients were diagnosed with HSIL or SCC. It’s noteworthy that of the 652 patients who were pathologically diagnosed with HSIL by CDB, the cytology results were less severe than or equivalent to LSIL.

**Table 1:** Characteristics of patients with LSIL and HSIL diagnosed by CDB.

Characteristics	Group	Number of participants		
		LSIL (*n* = 410)	HSIL (*n* = 1,086)	Total (*n* = 1,496)
Age, mean		41.43 ± 9.49	38.43 ± 9.43	39.38 ± 9.70
TZ	TZ1	95	380	475
	TZ2	141	419	560
	TZ3	174	287	461
HR-HPV infection	Negative	42	133	175
	Positive	368	953	1,321
	Single	272	701	973
	Multiple	96	252	348
Cytology results^a^	≤LSIL	318	652	970
	≥HSIL	15	218	233
Age of first sexual intercourse	≤20	58	249	307
	20–25	226	470	696
	≥25	51	150	201
Number of sex partners	1	232	526	758
	2	76	167	243
	≥3	28	145	173
Parity	0	21	151	172
	1	174	386	560
	≥2	38	114	152

TZ, cervical transformation zone; TZ1, type 1 cervical transformation zone; TZ2, type 2 cervical transformation zone; TZ3, type 3 cervical transformation zone. ^a^≤LSIL (NILM, negative for intraepithelial lesion or malignancy; ASC-US, atypical squamous cells of undetermined significance; ASC-H, atypical squamous cells cannot exclude HSIL; LSIL, low-grade squamous intraepithelial lesion); ≥HSIL (HSIL, high-grade squamous intraepithelial lesion; AGC, atypical glandular cells).

### Comparative analysis between CDB and LEEP pathology

To evaluate the diagnostic efficacies of CDB for cervical lesions, we compared the final diagnosis results between CDB and histopathological results (Table 2). Surprisingly, the overall concordance rate between the pathological results obtained from CDB and LEEP was 70.12% (1,049/1,496). The final diagnosis was determined based on the higher lesion grade identified in the pathological examination between CDB and LEEP. The correct diagnosis rate of CDB is 93.38% (1,397/1,496). Out of 410 patients diagnosed with LSIL by CDB, 93 cases were confirmed as chronic cervicitis, 238 cases as LSIL, and 79 cases as HSIL through postoperative pathology examination of LEEP. Upgraded cases were observed in 19.27% (79/410) of instances. Among 1,086 patients initially diagnosed with HSIL by CDB, 171 (15.75%), 416 (38.31%), and 890 (81.95%) patients were diagnosed with chronic cervicitis, LSIL, and HSIL by LEEP, respectively. Notably, 19 cases were confirmed as cervical cancer, comprising 16 squamous cell carcinomas (SCC) and 3 adenocarcinomas (AC). Additionally, 1 case was confirmed as adenocarcinoma *in situ* by postoperative pathology, with both cytology and biopsy results revealing HSIL. In total, histologic upgrade was observed in 98 patients, including 79 cases of LSIL and 19 cases of HSIL, with a statistically significant difference in the histologic upgrade rate between the LSIL and HSIL groups (*χ*^2^ = 149.22, *p* < 0.001).

**Table 2:** Comparison of pathological results between CDB and LEEP.

CBD histopathology	Total	LEEP histopathology						Final diagnosis agreed with CBD	Accuracy (%)
		Cervicitis	LSIL	HSIL	AIS	SCC	AC		
LSIL	410	93	238	79	–	–	–	331	80.73%
HSIL	1,086	77	178	811	1	16	3	1,066	98.16%
Total	1,496	171	416	890	1	16	3	1,397	93.38%

LSIL, low-grade squamous intraepithelial lesion; HSIL, high-grade squamous intraepithelial lesion; AIS, adenocarcinoma in situ; SCC, squamous cell carcinoma; AC, adenocarcinoma.

### Comparison of cytology with the final diagnosis

We conducted a comparison of 1,202 patients for whom cytologic results were obtained against the final diagnosis, and one patient with a final diagnosis of AIS was excluded from the analysis (Table 3). Among 267 cases classified as NILM by TCT, the final pathological diagnosis showed 56 cases (20.97%) were LSIL, 206 cases (77.15%) were HSIL, while 5 cases (1.87%) turned out to be cervical cancer. Of the total 915 patients who were ultimately diagnosed with HSIL, only 220 patients had TCT results that indicated HSIL, and 2 patients displayed AGC results. It is interesting to note that, out of the 15 patients diagnosed with cervical cancer, 10 patients had TCT results that were no more severe than HSIL.

**Table 3:** Comparison of cytologic results with the final diagnosis.

Cytology results	Final diagnosis		
	LSIL	HSIL	CC
NILM (*n* = 267)	56 (20.97%)	206 (77.15%)	5 (1.88%)
ASC-US (*n* = 372)	96 (25.81%)	274 (73.66%)	2 (0.53%)
ASC-H (*n* = 76)	13 (17.11%)	60 (78.95%)	3 (3.94%)
LSIL (*n* = 255)	100 (39.22%)	153 (60.00%)	2 (0.78%)
HSIL (*n* = 228)	3 (1.32%)	220 (96.49%)	5 (2.19%)
AGC (*n* = 4)	2 (50.00%)	2 (50.00%)	0

CC, cervical cancer; NILM, negative for intraepithelial lesion or malignancy; ASCUS, atypical squamous cells of undetermined significance; ASC-H, atypical squamous cells cannot exclude HSIL; LSIL, low-grade squamous intraepithelial lesion; HSIL, high-grade squamous intraepithelial lesion; AGC, atypical glandular cells.

### Analysis of related factors of histologic upgrade

All cases were classified into two groups based on whether the pathological results of CDB were upgraded compared with those of LEEP. Following this categorization, logistic regression analysis was utilized to uncover the factors correlated with pathological histologic upgrade.

Among the 410 patients diagnosed with LSIL by CDB, 79 exhibited biopsy histologic upgrades. Univariate regression analysis was conducted on the 490 patients (Table 4). Both HPV16 (*p* = 0.001, *OR* 95%CI [1.50, 4.34]) and HPV18 infection (*p* < 0.001, *OR* 95%CI [1.71, 6.58]) were identified as statistically significant factors relating to biopsy histologic upgrade according to univariate logistic regression. Single HR-HPV infection demonstrated a higher propensity of being histologic upgrade than an HPV negative result (*p* = 0.038, *OR* 95%CI [1.08, 12.07]). Moreover, our findings revealed that type 2 transformation zone was more likely to be histologic upgrade by CDB compared to type 1 transformation zone (*p* = 0.001, *OR* 95%CI [1.83, 10.16]). Similarly, type 3 transformation zone showed a higher propensity towards histologic upgrade by CDB compared to type 1 transformation zone (*p* = 0.006, *OR* 95%CI [1.40, 7.69]). Subsequently, a multivariate logistic regression was employed to analyze predictive factors including type of cervical transformation zone, HR-HPV infection status and HPV16/18 infection. The analysis revealed that HPV16 (*p* < 0.001, *OR* 95%CI [1.58, 5.02]), HPV18 (*p* < 0.001, *OR* 95%CI [2.11, 9.29]), and the type of cervical transformation zone (*p* < 0.05) were statistically significant. These are recognized risk factors contributing to histologic upgrade of CBD in LSIL patients. Among the 1,086 patients pathologically confirmed with HSIL via CDB, 19 were additionally diagnosed with cervical cancer. The 19 underestimated patients were all over 35 years old, and the average age of the underestimated group was larger than that of the non-underestimated group (*t* = 3.002, *p* = 0.003). None of the 19 upgraded patients was type 1 transformation zone, and type 3 transformation zone was more likely to be upgraded than type 2 transformation zone (*χ*^2^ = 8.83, *p* = 0.003). Each one of these 19 patients was infected with HR-HPV, with 14 patients evidencing infection by a single HPV subtype. There was no significant difference in the histologic upgrade rate of CDB between HPV single infection and multiple infection (*χ*^2^ < 0.001, *p* = 0.990). Among the 19 upgraded patients, 63.16% (12/19) tested positive for HPV16, and 31.57% (6/19) tested positive for HPV18. Univariate regression analysis was performed to explore the impact of HPV genotypes on the histologic upgrade of HSIL (Table 5). The results showed that HPV16 infection (*p* = 0.049, *OR* 95%CI [1.00, 6.58]), HPV18 infection (*p* < 0.001, *OR* 95%CI [2.50, 18.40]) were significant factors influencing the histologic upgrade of HSIL. And multivariate regression analysis yielded consistent results. The results indicate that HPV genotype may play a crucial role in the accurate detection and diagnosis of HSIL, HPV16 and HPV18 infections warrant closer attention in clinical practice.

**Table 4:** Logistic regression analysis of histologic upgrade in patients with LSIL by CDB.

Variables	Group	Univariate regression		Multivariate regression	
		*OR* (95% CI)	*p* value	*OR* (95% CI)	*P* value
Age (years old)	≤46	1.00 (Reference)			
	>46	0.62 [0.35, 1.12]	0.115		
TZ	TZ1	1.00 (Reference)		1.00 (Reference)	
	TZ2	4.31 [1.83, 10.16]	0.001	5.10 [2.09, 12.45]	<0.001
	TZ3	3.28 [1.40, 7.69]	0.006	4.18 [1.72, 10.14]	0.002
HR-HPV infection	Negative	1.00(Reference)		1.00(Reference)	
	Single	3.60 [1.08, 12.07]	0.038	2.48 [0.72, 8.54]	0.151
	Multiple	2.80 [0.78, 10.12]	0.117	1.38 [0.35, 5.38]	0.643
HR-HPV positive	HPV16	2.55 [1.50, 4.34]	0.001	2.81 [1.58, 5.02]	<0.001
	HPV18	3.36 [1.71, 6.58]	<0.001	4.42 [2.11, 9.29]	<0.001
	HPV31	0.58 [0.17, 2.00]	0.392		
	HPV33	1.10 [0.43, 2.80]	0.84		
	HPV35	2.12 [0.38, 11.80]	0.39		
	HPV39	0.56 [0.19, 1.63]	0.284		
	HPV45	0.84 [0.10, 7.26]	0.871		
	HPV51	0.34 [0.10, 1.15]	0.084		
	HPV52	0.70 [0.36, 1.33]	0.274		
	HPV53	0.24 [0.06, 1.03]	0.056		
	HPV56	0.31 [0.04, 2.43]	0.267		
	HPV58	1.84 [0.98, 3.43]	0.057		
	HPV59	0.41 [0.05, 3.26]	0.401		
	HPV66	1.05 [0.29, 3.81]	0.942		
	HPV68	0.58 [0.17, 2.00]	0.392		

**Table 5:** Logistic regression analysis of histologic upgrade in patients with HSIL by CDB.

Variables	Group	Univariate regression		Multivariate regression	
		*OR* (95% CI)	*P* value	*OR* (95% CI)	*P* value
Age (years old)		1.06 [1.02, 1.11]	0.004	0.97 [0.91, 1.03]	0.275
TZ	TZ1		0.993		0.992
	TZ2	0.24 [0.08, 0.66]	0.006	0.15 [0.04, 0.51]	0.002
	TZ3	1.00 (Reference)		1.00 (Reference)	
HR-HPV infection	Negative		0.996		
	Single	3.60 [1.08, 12.07]	0.99		
	Multiple	1.00 (Reference)			
HR-HPV positive	HPV16	2.57 [1.01, 6.58]	0.049	0.29 [0.11, 0.77]^*^	0.013
	HPV18	6.78 [2.50, 18.40]	<0.001	0.11 [0.04, 0.34]^*^	<0.001
	HPV33	1.92 [0.55, 6.70]	0.307		
	HPV39	2.91 [0.37, 22.86]	0.31		
	HPV45	2.32 [0.30, 18.03]	0.423		
	HPV53	1.59 [0.21, 12.25]	0.656		
	HPV58	0.57 [0.13, 2.48]	0.452		

* OR values were calculated with HPV 16/18-positive infections as the reference group.

## Discussion

Our study underscores the importance of correlating different diagnostic steps—CDB and LEEP—in the management pathway of cervical SIL. By identifying specific risk factors for CDB histologic upgrade, such as HPV16/18 infection and non-fully visible transformation zones, our findings provide actionable insights for clinicians. Our study showed that 93.38% (1,397/1,496) of cervical SIL were diagnosed by CDB successfully, highlighting its significant role in early detection of cervical lesions. Nonetheless, the limited sampling obtained during CDB only allows for superficial tissue collection, resulting in potential histologic upgrade. This study aimed to investigate the influence of risk factors on biopsy histologic upgrade in patients with cervical SIL. Additionally, we utilized a substantial cohort to uncover the association between HPV genotype and the likelihood of biopsy histologic upgrade. The majority of cervical SIL typically do not present any noticeable clinical symptoms or signs. If cervical SIL progresses to invasive cancer, the prognosis is often unfavorable. Therefore, accurate identification of cervical HSIL and early cervical cancer is significantly crucial. Colposcopy-directed cervical biopsy serves as a crucial method for diagnosing cervical lesions. Research has indicated that biopsies performed in four quadrants at 3, 6, 9, and 12 o’clock on the cervix yield a higher diagnostic rate than those conducted solely at the site of suspected lesions (12). In our study, we adopted a combined approach: besides obtaining biopsies from suspicious sites, we also collected samples from designated locations within the four quadrants.

In our study, the prevalence of HR-HPV infection among the patients was 88.30%, which aligns with previous research findings (13). The three most commonly detected HR-HPV genotypes were HPV16 (35.63%), HPV52 (17.25%), and HPV58 (16.38%), consistent with a retrospective study (14). HPV18, with an infection rate of 7.75%, ranked as the sixth most prevalent genotype. Previous studies have established that HPV infection is essential factors contributing to the development of cervical squamous intraepithelial lesions and cervical cancer. Notably, HPV16 and HPV18 are responsible for approximately 70% of cervical squamous cell carcinomas and about 90% of cervical adenocarcinomas (3, 15). Additionally, it’s worth noting that the pathological grading after LEEP surgery was lower than CDB in 348 patients (23.26%). This could result from complete lesion excision during cervical biopsy or endocervical curettage. Consequently, to ensure accuracy, the final diagnosis was determined based on the higher pathological assessment achieved either through CDB or LEEP.

We conducted an explicit analysis to examine the relationship between HPV genotypes and biopsy histologic upgrade. Our analysis identified a strong association between HPV16/18 infection and histologic upgrade. While this could be partly attributable to CDB sampling limitations (e.g., deeper or more localized lesions), it is crucial to consider that HPV16/18 are biologically more oncogenic and are associated with a higher prevalence and rapid progression of high-grade lesions. Therefore, the observed upgrade may not solely reflect a CDB-specific sampling error but could also be influenced by the intrinsic aggressiveness of these viral genotypes, potentially leading to faster disease progression even within our defined 3-month window or the development of small, focal high-grade lesions that are easily missed by biopsy. Our sensitivity analysis in the ≤6-week subgroup supports sampling error as a dominant, but likely not exclusive, factor. Our findings regarding HPV16/18 differ from those of Stoler et al. (16), who reported that these genotypes did not increase the risk of biopsy histologic upgrade. This discrepancy may be explained by evolving clinical contexts. Their study was conducted in the placebo arm of vaccine trials (2001–2003) with a different patient population and colposcopy referral patterns. Crucially, as the authors note, genotyping for non-16/18 HR-HPV was not available at that time, preventing a direct comparison of genotype-specific risks as performed in our contemporary cohort. Furthermore, differences in biopsy protocols and the definition of the reference standard may contribute to the contrasting results.

We concluded that type 2 transformation zone (TZ2) and type 3 transformation zone (TZ3) were more susceptible to histologic upgrade than type 1 transformation zone (TZ1) in cases of LSIL diagnosed by CDB. In addition, all the 19 upgraded cervical cancer patients had TZ2 and TZ3, and TZ3 was more likely to be underestimated than TZ2. For lesions within the cervical canal, it is challenging to fully expose the affected sites during CDB, making observation and sampling difficult. Furthermore, the detection rate of endocervical curettage is low, which increases the likelihood of misdiagnosis. Gustafson et al. (17) analyzed 102 patients with TZ3 and found that the HSIL detection rate was significantly higher in LEEP specimens than in biopsies, resulting in more than half of HSIL cases being underestimated in biopsies. Age has also been reported as a risk factor for histologic upgrade (18). Our results also indicate that in HSIL group, the age of the underestimated patients was higher than that of the patients who violated the histologic upgrade. This may be because estrogen levels gradually decrease with age, especially in perimenopausal women, leading to cervical atrophy and upward migration of the transformation zone. This makes it difficult to fully expose the lesions for examination, increasing the rate of histologic upgrade. A meta-analysis revealed that oral estradiol, intravaginal estradiol, and intravaginal misoprostol can be used to convert TZ3 to TZ1 or TZ2. Intravaginal misoprostol is well-tolerated and more feasible due to its availability and shorter treatment schedule when compared to oral or intravaginal estradiol (19).

In this study, we found that the type of transformation zones and HPV16/18 infection were significant risk factors associated with biopsy histologic upgrade. Given these results, special attention should be accorded to patients with the aforementioned high-risk factors during the process of a cervical biopsy. Previous research has demonstrated that the number of biopsy sites affects the accuracy of cervical biopsy diagnosis (16, 20). A decrease in the number of biopsy sites correlates with an increased likelihood of histologic upgrade. Therefore, we can aim to increase either the number of biopsies or the depth of the biopsy sites to enhance the precision of colposcopy, particularly in cases with the aforementioned high-risk factors. Alternatively, in a case with a CDB diagnosis of LSIL for more than 1 year, we may consider cervical conization if it has HPV16/18 infection and type 2 and type 3 cervical transformation zones. It has been reported that implementing conization before carrying out radical hysterectomy yields improved survival rates and decreased recurrence rates for patients suffering from early-stage cervical cancer, especially those opting for minimally invasive surgery (21). Consequently, patients suspected of having cervical cancer based on colposcopy findings may proceed directly to cervical conization. Studies have further indicated that cervical lesions infected with HPV16 and 18 are susceptible to recurrence post cervical conization (22). Therefore, patients with HPV16/18 infection should be subject to close postoperative follow-up. For patients with a type 3 transformation zone, placement of vaginal misoprostol can help better visualize the cervical transformation zone, thereby enhancing the accuracy of biopsy.

Our study has limitations inherent to its retrospective design. We were unable to account for several potential technical confounders in our regression models, such as the precise number of biopsy fragments taken or individual colposcopist experience, as these data were not consistently recorded in the medical records. Furthermore, the analysis did not stratify the risk of upgrade based on the severity of the antecedent cytology result. These factors could influence the likelihood of histologic discrepancy and represent important avenues for future prospective studies with standardized data collection protocols. In addition, while we included only patients who underwent LEEP within 3 months of CDB to minimize the risk of disease progression, the lack of exact procedural dates precluded a sensitivity analysis using a standardized shorter interval (e.g., ≤6 weeks). Future prospective studies should record exact dates to allow for more granular time-to-event analyses, which would help further distinguish sampling error from rapid progression.

In conclusion, our study comprehensively analyzed the cervical biopsy pathology and postoperative LEEP pathology of 1,496 patients with cervical squamous intraepithelial lesion. Our findings indicate that HPV16/18, as well as cervical transformation zones, especially type 3 transformation zone, represent significant contributing factors to histologic upgrade resulting from colposcopy biopsy procedures. The comparative analysis between CDB and LEEP pathology in this large cohort allows us to move beyond a one-size-fits-all approach. The identified risk factors for histologic upgrade create a framework for personalized medicine in cervical precancer management. Integrating these factors into clinical decision-making can help tailor the diagnostic and therapeutic intensity to the individual patient’s risk profile. This targeted strategy, aiming for precise diagnosis and appropriate management, is a fundamental step towards improving healthcare efficiency and patient quality of life in the context of cervical cancer prevention.

## Data Availability

The raw data supporting the conclusions of this article will be made available by the authors, without undue reservation.
